# Adolescents' wellbeing and functioning: relationships with parents' subjective general physical and mental health

**DOI:** 10.1186/1477-7525-7-100

**Published:** 2009-12-15

**Authors:** George Giannakopoulos, Christine Dimitrakaki, Xanthi Pedeli, Gerasimos Kolaitis, Vasiliki Rotsika, Ulricke Ravens-Sieberer, Yannis Tountas

**Affiliations:** 1 Centre for Health Services Research, Department of Hygiene and Epidemiology, University of Athens Medical School, 25 Alexandroupoleos street, 115 27 Athens, Greece; 2 Department of Child and Adolescent Psychiatry, Athens University Medical School, "Agia Sophia" Children's Hospital, Athens, Greece; 3 Department of Psychiatry, Community Mental Health Center Byron-Kesariani, University of Athens, Athens, Greece; 4 Robert Koch Institute, Child and Adolescent Health, Berlin, Germany

## Abstract

**Background:**

This study aimed at examining the relationship between parental subjective health status and adolescents' health-related quality of life (HRQoL) as well as the role of gender, socioeconomic status, presence of chronic health care needs and social support on the above interaction.

**Methods:**

Questionnaires were administered to a Greek nation-wide random sample of adolescents (*N *= 1 194) aged 11-18 years and their parents (N = 973) in 2003. Adolescents' and parents' status was assessed, together with reports of socio-economic status and level of social support. Various statistical tests were used to determine the extent to which these variables were related to each other.

**Results and Discussion:**

Parental subjective mental health status was significantly correlated with adolescents' better physical and psychological wellbeing, moods and emotions, parent-child relationships, school environment and financial resources. Parental subjective physical health status was strongly associated with more positive adolescents' self-perception. Adolescents' male gender, younger age, absence of chronic health care needs, high social support, and higher family income were positively associated with better HRQoL.

**Conclusions:**

This study reinforces the importance of parental subjective health status, along with other variables, as a significant factor for the adolescents' HRQoL.

## Background

There is a substantial body of research which suggests the impact of parental factors on adolescents' development. Such factors include antenatal exposures, environmental and genetic determinants, parental behaviours [1], socioeconomic status [2-4], family histories of psychopathology [5-7], marital and family conflict [8,9] and the parent-adolescent relationship [10-12]. The physical, social, emotional, and educational outcomes for adolescents are highly dependent on experiences within their family [13].

However, health professionals pay little attention to adolescents' experience of parental illness generally. Adolescents' feelings and emotional reactions to the physical and mental alterations that the illness may impose to a parent are often neglected. Adolescents, especially the younger ones, often find it difficult to understand the causes of the abrupt changes in family interrelationships due to parental illness and/or to cope with the considerable family discordance and the possible demands to undertake extended duties and new roles inside the family [14]. Even less information exists as to how parental general health status is associated with adolescents' health related quality of life (HRQoL) - a significant health outcome measure in clinical and epidemiologic studies nowadays [15,16]. The concept of HRQoL reflects a subjective, multidimensional and comprehensive model of health concerned with dimensions such as physical and psychological well-being, family life, school performance and peer relations [17].

Quality of life (QoL) is a complicated concept that is difficult to define and measure [18]. It is often used as a synonym for happiness including agents that contribute to the wellness and meaning of life. QoL is understood to be the personal satisfaction with the cultural or intellectual conditions under which an individual lives. QoL is a broad concept having relevance to almost all areas of human function. As a result, it has been extensively researched, reviewed, and discussed in the social science, psychology, economic, and medical literature. However, one of the important domains of QoL is health. Health can also be viewed as a subjective representation of function and well-being, which is not only understood by somatic indicators, but comprises how a person feels, psychologically and physically, and how she or he manages with other persons and copes with everyday life. Health Related Quality of Life (HRQoL) is described as a multidimensional construct covering physical, emotional, mental, social, and behavioural components of well-being and function as perceived by patients and/or other individuals [19]. Moreover, HRQoL can reflect an individual's perception of their position in life in the context of the cultural and values systems in which they live and in relation to the goals, expectation, standards and concerns. The assessment of HRQoL is, thus, related to broad social and public health concerns and can offer potential applications for need assessment and social policy formulation.

The definition of HRQoL used for adults could be applied to adolescents, although specific aspects of physical development and psychosocial functioning as well as distinct features of adolescence as opposed to childhood and adulthood should be considered [20]. Only a few, but an increasing number of generic questionnaires exist which assess HRQoL in children and adolescents. This has to do first with doubts as to whether children and adolescents can reliably express opinions, attitudes and feelings about their HRQoL and secondly with the relative absence of reliable and valid measures. The age, maturity and cognitive/emotional development of the child/adolescent should be taken into consideration in any effort to measure the concept of HRQoL. Recent research has shown that children are able to report on their well-being and functioning reliably if the questionnaire is appropriate to the child's age and cognitive level [21]. Adolescents are not regarded as small adults, their special health needs should be acknowledged. Adolescents are growing in the various social environments including family, school, peers, neighbourhoods, and community [22]. On the contrary to adults, they often cannot make any alterations to disadvantageous environment. Moreover, their growth and maturation necessitates the longitudinal evaluation of HRQoL in different time points of development. Additionally, the sense of self and the need for independence are valued as important as physical functioning, general mood and social relationships among adolescents [23]. In fact, despite the increasing importance of peers in adolescence, family relations maintain a central role in adolescent life satisfaction [24,25].

Research in the interconnection between parental health and adolescents' functioning is mainly limited to studies with small numbers of adolescents or parents with specific illnesses rather than general population health surveys or health outcome research. Somatic illness in a parent is a risk factor for psychiatric disorder in adolescents [26,27]. Moreover, some studies have shown that the presence of a significant somatic disease in a parent effects on adolescents' development and psychosocial functioning [28,29] and the diagnosis of a severe physical disease may be a major life-changing event for both patients and their children [30,31]. Additionally, research has suggested a close interrelation among developmental difficulties in the child and progress of parental chronic illness within the family life cycle [32]. However, other studies concluded to contradictory results (i.e. there is no significant association between characteristics of parental physical ill health and adolescent functioning) suggesting the unclear impact of parental health status on adolescents' wellbeing and functioning [33].

Ample evidence is available regarding the association between parental mental disorder and adolescents' poor adjustment [34]. It has been reported that the quality of interpersonal relationships within the family mediates mostly this association [35,36]. The parental disorder may limit the adolescent's identification with their parents and/or the parent may be unable to help the adolescent acquire competence and develop independence/autonomy. Adolescents' socio-psychological adjustment can be also at risk due to marital conflicts and problematic parenting practices characterizing families with parental mental health problems. The borders between generations can be diffused and the adolescent may be engaged in parental problems and conflicts [37]. Various studies have showed that problems extend beyond family life boundaries to low school performance and problems in peer relations [35,38,39].

It should be noted that a number of factors, such as the parent's and adolescent's gender, the adolescent's age, the socio-economic status of the family and the presence of a chronic illness on the adolescent, may facilitate or impede positive adjustment to parental poor health. From available literature, it appears that children facing a chronic illness and those coming from low income families, older adolescents, and girls more than boys, are at higher risk for multiple problems when parents -especially mothers - fall ill [1,34,38]. Moreover, research examining the ways in which families are able to continue to meet their children's developmental needs, despite the presence of physical illness, suggests the important role of social support networks, as a major benefit for adolescents' resilience (i.e. the assets and resources that enable some adolescents to overcome the negative effects of risk exposure [33].

The aim of the present paper was to extend previous research by examining the relationship between parental subjective physical and mental health and adolescents' reporting of their HRQoL in a general population. Parental subjective physical and mental health here is approached in terms of everyday functioning and wellbeing rather than of a specific diagnosed illness. This study also sought to determine whether relationships observed between parental subjective general health status and adolescents' HRQoL are similar across different domains of adolescents' wellbeing and functioning (e.g. physical, psychological, social aspects of HRQoL). Specifically, the present study investigated the relationship between parental subjective general health and adolescents' HRQoL on the basis of the following hypotheses: 1) Parental subjective health variables, i.e. self-perceived physical and mental health, will be positively associated with the level of adolescents' HRQoL, and 2) Age, gender, family socio-economic status, the presence of chronic health care needs in the adolescent and social support will be significant factors in the interrelationship between parental subjective health status and adolescents' HRQoL, with older girls of low income family background, with more chronic health care needs, and less social support, reporting poorer HRQoL.

## Methods

### Participants and Procedures

The study was conducted during the year 2003 in Greece within the framework of the European project "Screening and Promotion for HRQoL in Children and Adolescents - A European Public Health Perspective" [17]. The sampling was random, multi-staged and based on the age and sex distribution of school children living in the 54 geographical sectors of the country, according to data from the National Census of 2001 [40]. Schools in each sector were randomly selected by a computer program and students of each selected school were selected randomly from classroom name lists. A sample of 1,900 adolescents (11 to 17 year olds) was recruited. Adolescents filled in the questionnaire at school. A total of 1,194 (i.e. 63% response rate) of self-reported questionnaires (40.07% boys) were returned. Inclusion criteria for the adolescents were to be between 11 and 18 years old, to be able to read and complete the questionnaires themselves, and to consent to be involved in the study. Adolescents took parent surveys home. Parents were asked to complete the questionnaire at home and return it back to school within a week time-limit. Inclusion criteria for the parents were to live with the adolescent. Only one parent was involved for each adolescent included in this study. Each family was free to select which parent responded. The adolescent and the parent completed the questionnaire sequentially. The study involved 973 families.

### Measures

#### Adolescent's status

Adolescents' HRQoL was measured using the KIDSCREEN-52, a generic self-reported questionnaire for children and adolescents from 8 to 18 years with good psychometric properties [17]. It is intended to assess HRQoL from the child's/adolescent's perspective and focus on physical, mental and social dimensions of wellbeing. The KIDSCREEN instrument aims at identifying children and adolescents at risk with regard to their subjective health. It includes ten HRQoL dimensions: 1) physical wellbeing; 2) psychological wellbeing; 3) moods and emotions; 4) self-perception; 5) autonomy; 6) parent relations and home life; 7) social support and peers; 8) school environment; 9) social acceptance and bullying; and 10) financial resources. The KIDSCREEN-52 HRQoL questionnaire assesses either the frequency of behaviour/feelings or, in fewer cases, the intensity of an attitude. Both possible item formats use a 5-point Likert response scale, and the recall period is 1 week. Total score from each dimension is ranging from 0 to 100, with higher scores indicating higher HRQoL. The Greek version of the instrument has been found to have good reliability with Cronbach's α for its 10 dimensions ranging satisfactorily between .76 (Bullying) - .89 (Financial Resources). Convergent and discriminatory validity, tested against information about the adolescents' physical and mental health have also been found at satisfactory levels [17]. The KIDSCREEN-52 version for adolescents was used in the present study.

To assess special chronic health care needs, the Children with Special Health Care Needs Screener was included in the proxy questionnaire, as measure of adolescents' physical chronic health status [41]. The CSHCN contains five question sequences: each question is followed by two additional questions, asking about the presence and duration of any health conditions. The five questions address the use or need of prescription medication; the use or need of medical, mental health or educational services; functional limitations; use and need of specialized therapies (occupational therapy, physiotherapy, speech therapy, etc.); and treatment or counselling for emotional or developmental problems, all associated with a health problem that has lasted or is expected to last 12 months or longer. The CSHCN screener results were combined and recorded in a binary variable (positive versus negative result) for the analysis purposes.

#### Parent's status

Parental subjective health status was assessed with the use of the self-administered the SF-12 questionnaire (Greek standard version 1.0). The 12-item Health Survey (SF-12) was developed as a shorter version of the SF-36 for use in large-scale studies, particularly when overall subjective physical and mental health are the outcomes of interest instead of the typical eight domains of the extended measure (i.e. physical functioning, role physical, bodily pain, general health perception, vitality, social functioning, role emotional, and mental health). The Greek SF-12 is a brief, yet valid, alternative to the SF-36 [42]. The domain scores were chosen to be used in the present analysis and were scale data of 0-100 and the summaries were deviation scores of mean 50. Missing values were treated according to procedures suggested in the SF-12 manual [43].

#### Socio-economic status & level of social support

To assess familial socioeconomic status the Family Affluence Scale (FAS; Currie, Elton, Todd & Platt, 1997) was used, addressing issues of family car ownership, having their own unshared room, the number of computers at home and times adolescents spent on holiday in the past 12 months. The FAS was collected in seven categories (from 0 the lowest, to 7 the highest FAS category) and was re-coded into three groups in the analysis (low FAS level (0-3), intermediate (4-5) and high FAS level (6-7)). The psychometric properties of the FAS are acceptable and support its use as a self-reported adolescents' measure [44]. To assess the level of social support, the Oslo 3-Item Social Support Scale was adapted [45]. This scale contains one question about the number of people who can provide a sense of security and support to the adolescent and two questions about emotional and instrumental support from those people. The total score calculated by summarising those three items ranged from 0 to 11 with values less than 6 recognised in the literature as "poor social support" [46].

### Statistical Analysis

Adolescents' HRQoL was assessed through the KIDSCREEN-52 HRQoL questionnaire. Its ten dimensions (total score for each one ranging from 0 to 100) formed the outcome measures of the present study. The two component summary scales (physical and mental) of the SF-12 questionnaire were used as indicators of the parental subjective health status, i.e. the main explanatory variables under study. Demographic characteristics such as adolescent's age and gender, familial socio-economic status, level of social support as well as the adolescent's physical health status were considered as potential covariates. All analyses were performed with STATA software, version 8.2.

Exploratory data analysis includes the calculation of descriptive statistics for all outcome variables and covariates. Continuous variables are summarized through means and standard deviations while for categorical variables absolute and relative frequencies are given. Investigation of the relationships between outcome variables and covariates was performed in a two-step process. Firstly, all bivariate associations were assessed with the use of different statistical tests, according to the nature of the variables examined. More specifically, Student's t-test was used to compare the distribution of a specific HRQoL dimension between the levels of a binary variable and analysis of variance was performed for categorical variables with more than two levels. Pearson correlation coefficients were calculated and univariate linear regression models were fitted to assess the bivariate relation of continuous variables. After examining all the bivariate relationships, multiple linear regression models were employed to determine the set of covariates that best explain children's HRQoL, as measured by each dimension of the HRQoL questionnaire separately. The groups of candidate variables for entering each model were consisted of covariates with *p *< .20 in the corresponding univariate analyses. A backward stepwise process was used for the inclusion of candidates in each regression model. The significance levels for addition and removal from the model were fixed to .05 and .10 respectively. So variables with *p *< .05 were eligible for inclusion and variables with *p *≥ .10 were eligible for exclusion from the model. Because of different percentages of missing data in the recorded variables, the number of observations in the multiple regression models varies from 903 (model for financial resources) to 1 162 (model for autonomy).

Due to the bounded and skewed distributions of the KIDSCREEN scores in order to check the robustness of results, analysis was repeated using non-parametric analogues and the results (not presented here) were similar to those obtained by the conventional parametric analysis. With regard to the agreement between the results obtained by the two approaches and the established methodology in subject-related researches [47], we adopt and present findings from a parametric statistical approach [48].

## Results

### Features of the study population

A sample of 1,900 adolescents (11 to 17 year olds) was recruited. A total of 1,194 KIDSCREEN-52 self-reported questionnaires and 1,187 SF-12 questionnaires were returned (that is, approximately 63% response rate). The sample of the analysis consisted of 1 194 adolescents, 40.07% male and 59.93% female, of mean age 14.66 (± 1.73) years old and one parent for each adolescent (Table 1). Response rates for each KIDSCREEN-52 HRQoL dimension ranged from 80% to 100%. About 88% of the participant parents gave information about their gender. According to the provided information, the parent surveys were mostly filled by mothers (76.12%). The total KIDSCREEN scores demonstrate distributions similar to those found by previous studies [17]. Scores of PCS and MCS, the two measures of parental health status, had mean values of 47.83 ± 6.16 and 50.24 ± 8.81 respectively. The CSHCN question sequences revealed existence of special health care needs in 3.36% of the participants. The majority of families participating in the sample (44.96%) were classified as belonging to the intermediate level of the family affluence level, and the mean for the OSLO social support sum score was 11.06 ± 1.86, range 3 to 14.

**Table 1:** Features of the Study Population

Variable	N (%)	Mean ± SD (range) *(Unless specified otherwise)*
Age	1 194 (100.0)	14.66 ± 1.73 (10-21)
Adolescent's Gender*	1 193 (99.91)	
Male		715 (59.93%)
Female		478 (40.07%)
Participant Parent's Gender*		
Mother		797 (76.12%)
Father		244 (23.30%)
Other		6 (0.57%)
Physical Well-being	1 178 (98.66)	66.11 ± 19.16 (0-100)
Psychological Well-being	1 187 (99.41)	70.03 ± 19.35 (0-100)
Moods & Emotions	1 168 (97.82)	72.64 ± 18.22 (0-100)
Self Perception	1 181 (98.91)	66.44 ± 21.00 (0-100)
Autonomy	1 173 (98.24)	58.69 ± 23.55 (0-100)
Parents Relations & Home Life	1 168 (97.82)	70.46 ± 20.19 (0-100)
Peers & Social Support Relations	1 161 (97.24)	70.37 ± 21.27 (4.17-100)
School Environment	1 172 (98.16)	64.24 ± 18.74 (0-100)
Bullying	1 188 (99.50)	91.87 ± 14.03 (0-100)
Financial Resources	1 184 (99.16)	69.52 ± 24.33 (0-100)
Parental Health Status (PCS)	973 (81.49)	47.83 ± 6.16 (17.47-62.68)
Parental Health Status (MCS)	973 (81.49)	50.24 ± 8.81 (11.11-69.18)
Results for CSHCN screener*		
Yes	1 043 (87.35)	35 (3.36%)
No		1,008 (96.64%)
How well-off do you think the adolescent's family is?*		
Very well	1 017 (85.18)	65 (6.39%)
Quite well		272 (26.75%)
Average		584 (57.42%)
Not very well		84 (8.26%)
Not at all well		12 (1.18%)
Social class of the adolescent's family*		
Lower	965 (80.82)	4 (.41%)
Working		161 (16.68%)
Middle		501 (51.92%)
Upper middle		238 (24.66%)
Upper		38 (3.94%)
None		23 (2.38%)

* Absolute and relative frequencies are given.

### Bivariate analysis

Bivariate associations are summarized in Additional file 1. A standard group of variables consisted from MCS, age and gender of the adolescent, FAS and OSLO social support sum score is significantly associated with adolescent's psychological well-being, moods and emotions, parents relations and home life, and peers and social support relations. Adolescent's physical well-being is also marginally associated with CSHCN screener result while self perception is univariately affected by all the recorded variables except for the CSHCN screener result. Male children reported better scores for their autonomy than female adolescents. This dimension of the KIDSCREEN-52 HRQoL questionnaire is also negatively associated with age, and positively associated with OSLO social support sum score. Its positive relation to MCS is marginally significant at the 5% significance level. Scores indicating the HRQoL aspect of social acceptance and bullying are better (higher) for female adolescents compared to male adolescents and for adolescents with a negative CSHCN screener result compared to a positive result (marginally significant association). Bullying is also significantly affected by the OSLO social support sum score. The last dimension of the KIDSCREEN-52 HRQoL, namely, financial resources has a significant association with MCS, FAS and OSLO social support sum score and a marginally significant association with the adolescent's gender. The specific mean score is higher for males than females and for adolescents included in the higher level of the family affluence scale compared to the intermediate and lower levels.

The effect of the PCS and MCS on the dimensions of the KIDSCREEN-52 HRQoL was also assessed separately for males and females. Analysis by gender showed that females' HRQoL is significantly affected by PCS only with regard to self-perception (Pearson r = 0.11, p = 0.0077) while neither dimension of males' HRQoL is significantly correlated with PCS. Contrarily, MCS has a significant association with the vast majority of the dimensions describing females' HRQoL. In specific, it was found that social acceptance and bullying is the only dimension which is not significantly affected by MCS between girls. The effect of MCS on males' HRQoL is less evident since it is significantly associated only with two dimensions: physical well-being (Pearson r = 0.12, p = 0.0144) and financial resources (Pearson r = 0.17, p = 0.0009).

### Multivariable analysis

Variables significant in the model for adolescent's physical well-being were mental health of parents (MCS), age and gender of the adolescent, the CSHCN screener result and the level of social support as expressed by the OSLO social support sum score (Additional file 2). The adjusted *R*^2 ^was equal to .19. The total KIDSCREEN-52 score for the physical well-being increases by 2.49 points with one point increase on the OSLO social support scale, indicating the importance of social support for the adolescent's quality of life. Every point increase on the MCS scale is associated with a .26-point increase in the physical well-being score. Even if this effect is not so strong it demonstrates a close, positive association between parents' mental health and child's physical health. Higher scores are also reported by younger adolescents, males compared to females and adolescents with negative CSHCN screener results compared to adolescents with positive results.

The final model for adolescent's psychological well-being includes as covariates the parental mental health status (MCS), age and gender of the adolescent and the OSLO social support sum score. Adolescents younger by one year of age report scores higher by 1.99 units in average compared to one year older adolescents. This result could be rather due to a limited self-awareness in younger ages. The self-report of the score for the specific dimension of the KIDSCREEN-52 HRQoL questionnaire is increased by 5.09 units for males compared to females, revealing a better psychological health status for the male gender. MCS and OSLO social support sum score have also a positive effect. The adjusted *R*^2 ^equals .21. The same set of covariates is included in the models for moods and emotions and for parent's relations and home life. Relationships keep the same directions and the adjusted *R*^2 ^are .26 and .27 respectively.

Multivariable analysis for adolescent's self-perception total score resulted in a model consisted of parental physical and mental health status (PCS, MCS), age and gender of the adolescent and OSLO social support level (*R*^2^-adjusted = .18). Every 10-points increase on the PCS scale is associated with a 3.20-point increase in the total score for self-perception, a finding that indicates the manifold effects of parents' physical health status. Male adolescents reported higher by 10.57 units scores compared to female adolescents, age had a negative effect and OSLO social support sum score was positively correlated with the self-perception total score reported.

Total score for adolescent's autonomy is affected by the age and gender of the adolescent and OSLO social support sum score (*R*^2^-adjusted = .12) while the final model fitted for peers and social support relations includes only two covariates; gender and OSLO social support sum score (*R*^2^-adjusted = .18). In the linear regression model for school environment, MCS, age, the OSLO social support sum score and the CSHCN screener result are included. Positive relationships were demonstrated with MCS and OSLO social support sum score, while the effect of age proved negative. Adolescents with a negative result from CSHCN screener reported scores higher by 7.06 units compared to adolescents with a positive result. Thus problems associated with a poor physical chronic health status are more serious and apparent in the broad community than inside home. This is an expected result since social acceptance and social support are two concepts closely related. The adjusted *R*^2 ^of the model was .19.

Self-reported scores for bullying were lower for males than for females and for adolescents with a positive CSHCN screener result than for adolescents with a negative result. Also, every point increase on the OSLO social support scale is associated with a 2.93-point increase in the total score for social acceptance and bullying. The adjusted *R*^2 ^for this model is .10.

Parental subjective mental health status (MCS), familial socio-economic status as described by the family affluence scale (FAS) and the level of support assessed through the OSLO social support sum score formed the set of covariates that best explain adolescent's perception of his/her financial resources. According to the fitted model (*R*^2^-adjusted = .22), adolescents included in the intermediate FAS level, report scores higher by 10.70 units in average than adolescents in the lower FAS level. The difference is even higher for adolescents belonging to the higher FAS level; this group reports financial resources scores that are by 16.93 units higher compared to adolescents in the lower FAS level. It turns out that adolescents seem to have a full sense of their families' financial status. The MCS and OSLO social support sum score are also positively associated with the self-reported scores of the specific dimension.

## Discussion

The aim of the present study was to explore the relationship between parental subjective physical and mental health and adolescents' HRQoL across various dimensions of everyday wellbeing and functioning. Additionally, an objective of this investigation was to examine the possible role that other factors (gender, socioeconomic status, presence of chronic health care needs and social support) may play in the above interaction. In general, there were some significant associations between parents' reports of their own subjective health and adolescents' reports of their HRQoL. The reporting of low subjective physical health status by parents was strongly associated solely with reporting less positive self-perception by adolescents, among the ten dimensions of adolescent HRQoL. It should be stressed that the dimension of self-perception here measures whether the appearance of the adolescent's body is viewed positively or negatively and reflects the value somebody assigns to him/herself and the perception of how positively others value him/herself. This finding is consistent with previous studies on children of somatically ill parents [26]. The low self-perception of adolescents may evolve through identification with the parent perceiving that his/her physical health - and possibly his/her physical appearance and body image - is distorted. The present finding that parental subjective physical health status was not significantly associated with any other adolescent HRQoL dimension is also supported by previous studies stressing the unclear impact of parental physical ill health on child functioning [33]. Moreover, the lack of association between parents' subjective physical health status and adolescents' HRQoL may be attributed to methodology issues. First, the use of a generic measure for examining the HRQoL among adolescents may not easily detect the impact of parental subjective health status on adolescents' wellbeing and functioning. Second, population-based samples are often unable to detect associations since clinical disorders and severe illnesses are rather rare and families from low socioeconomic background (where the associations may be stronger) are underrepresented.

Stronger associations were found between parents' subjective mental health status and adolescents' HRQoL. Better parental subjective mental health status was found to be significantly correlated with higher physical and psychological wellbeing, moods and emotions, parent-child relationships, school environment and financial resources. It is noteworthy that parental subjective mental health status is associated with multiple dimensions of adolescent wellbeing and functioning. These results are consistent with previous studies and emphasize how diverse are the effects that parental mental health concerns may have on child health, functioning and adjustment [34].

Regarding other significant factors in the interplay between parents' and adolescents' wellbeing, our study confirmed that mainly male gender, younger age and social support as well as absence of chronic health care needs to a lesser extent are positively associated with high children's HRQoL in various domains, so as to assume that all these factors can favor the positive adjustment of children to poor parental health or they are able to protect children from the adverse effects of low parental wellbeing on children's status. It is noteworthy that gender, age and social support seem to be associated more strongly than parental health status with adolescents' HRQoL. The role of social support, in particular, is highlighted since social support is strongly related to the majority of HRQoL dimensions and is a factor which can be enhanced through proper interventions in the direction of improving children's resilience towards parental illness, distress or lack of quality of life.

The strengths of the present study were the large and representative sample size from a general population of adolescents and their parents. The use of comprehensive measures of child HRQoL and parent functional health and well-being enabled the analysis of the parent and child health from a more contemporary perspective (i.e. health is regarded as a subjective and multidimensional human state) than only the absence of illness or disease [1]. In particular, unlike most of the previous research on the topic which has used information from already clinically diagnosed cases of parents, or conducted clinical assessments on parental general populations, the present study investigated the issue from a more public health perspective by collecting information on parental self-perceived general health status. Positively focused measures can enable the measurement of the full spectrum of psychological wellbeing rather than requiring researchers to infer positive constructs from the absence of negative indicators.

As the study was cross-sectional, it was not possible to assess whether there was a causal relationship between parental subjective health status and adolescents' HRQoL. Also, the association of adolescents' HRQoL with parental subjective health status according to parental gender was not examined, since only small numbers of fathers responded and each family was free to select which parent responded. Moreover, only one response was obtained from each family, preventing comparisons of reports from both parents on the one adolescent. Gender differences deserve further research.

## Conclusions

This study lends further support to previous research that has addressed the impact of parental subjective health status as a significant factor for the adolescents' perception of how well they feel and function. The associations observed in this paper suggests the importance of addressing the issue of parental subjective health status when clinicians, counselors, educators and researchers have to understand and/or treat problems of adolescents' low wellbeing and functioning in new models of health promotion and care. Great attention should be given to addressing parental mental health issues through targeted psychosocial individual and/or family therapies when detecting a probable mental health problem in adolescent populations. However, professionals should take into consideration several factors such as adolescents' age, gender, perceived social support and chronic health care needs in order to assess effectively and manage the impact of parental health on adolescents' HRQoL.

## Competing interests

The authors declare that they have no competing interests.

## Authors' contributions

GG, CD, XP, GK and VR participated in the preparation of the paper. UR-S coordinated the European project "Screening and Promotion for HRQoL in Children and Adolescents - A European Public Health Perspective". YT had the overall supervision of the present study. All authors read and approved the final manuscript.

## Supplementary Material

Additional file 1 Bivariate associations of the dimensions of the KIDSCREEN-52 questionnaire with parental health status and socio-demographic characteristics Click here for file

Additional file 2 Multivariate analyses of the KIDSCREEN-52 questionnaire's ten dimensions Click here for file
