# Supplementary material for: Adolescents' wellbeing and functioning: relationships with parents' subjective general physical and mental health
Source: Health Qual Life Outcomes. 2009 Dec 15;7:100. doi: 10.1186/1477-7525-7-100 (PMC2804705; doi:10.1186/1477-7525-7-100)
Supplement: Additional file 1 — Bivariate associations of the dimensions of the KIDSCREEN-52 questionnaire with parental health status and socio-demographic characteristics [file 1477-7525-7-100-S1.DOC]

**Bivariate associations of the dimensions of the KIDSCREEN-52 questionnaire with parental health status and socio-demographic characteristics**

|  | **Dimensions of Adolescents’ HRQoL (Mean scores (standard deviations) unless specified otherwise)** | | | | | | | | | |
| --- | --- | --- | --- | --- | --- | --- | --- | --- | --- | --- |
| **Variables** | Physical Well-being | Psychological Well-being | Moods and Emotions | Self Perception | Autonomy | Parent Relations and Home Life | Social Support and Peers | School Environment | Social Acceptance and Bullying | Financial Resources |
| PCS^§^ | .02 | .00 | .02 | .11^†^ | -.04 | .03 | -.03 | .06 | .05 | .05 |
| MCS^§^ | .18^*^ | .19^*^ | .16^*^ | .12^†^ | .06 | .15^*^ | .10^†^ | .16^*^ | .01 | .20^*^ |
| Age^§^ | -.25^*^ | -.23^*^ | -.27^*^ | -.12^*^ | -.22^*^ | -.23^*^ | -.09^†^ | -.31^*^ | .03 | -.05 |
| *Gender* |  |  |  |  |  |  |  |  |  |  |
| Male | 71.45 (17.85) | 73.71 (16.90) | 76.89 (15.84) | 72.54 (19.80) | 63.07 (23.35) | 73.23 (18.87) | 72.05 (20.14) | 63.93 (19.71) | 90.40 (16.77) | 71.06 (23.48) |
| Female | 62.54 (19.18)^*^ | 67.60 (20.47)^*^ | 69.82 (19.14)^*^ | 62.33 (20.79)^*^ | 55.83 (23.22)^*^ | 68.63 (20.85)^*^ | 69.26 (21.92)^†^ | 64.43 (18.10) | 92.83 (11.79)^†^ | 68.54 (24.83) |
| *Family affluence scale* | |  |  |  |  |  |  |  |  |  |
| Low | 62.82 (19.16) | 67.19 (19.69) | 69.55 (18.78) | 64.07 (21.31) | 57.16 (24.01) | 66.49 (21.06) | 67.45 (22.34) | 61.50 (19.09) | 91.63 (14.43) | 60.14 (25.44) |
| Medium | 66.75 (18.73) | 71.59 (18.47) | 74.12 (17.42) | 67.07 (20.76) | 59.53 (23.98) | 72.57 (19.12) | 71.34 (20.16) | 65.69 (18.25) | 92.13 (13.68) | 73.41 (21.56) |
| High | 70.58 (18.08)^*^ | 70.47 (19.97)^†^ | 73.90 (18.65)^†^ | 68.49 (21.22)^†^ | 58.60 (21.62) | 72.65 (19.95)^*^ | 72.38 (21.08)^†^ | 66.38 (18.18)^†^ | 91.80 (13.87) | 81.10 (19.80)^*^ |
| *Results from CSHCN screener* | | |  |  |  |  |  |  |  |  |
| Negative | 58.82 (23.22) | 70.48 (19.66) | 71.73 (16.53) | 62.71 (19.75) | 63.57 (23.38) | 67.40 (24.22) | 74.64 (18.86) | 56.19 (22.85) | 85.00 (22.76) | 67.86 (24.52) |
| Positive | 66.17 (19.03) | 69.99 (19.26) | 72.48 (18.47) | 66.52 (21.13) | 58.11 (23.65) | 70.34 (20.03) | 70.10 (21.48) | 64.33 (18.41)^†^ | 92.17 (13.74) | 69.91 (24.35) |
| OSLO social support^§^ | .29^*^ | .39^*^ | .40^*^ | .32^*^ | .26^*^ | .48^*^ | .42^*^ | .34^*^ | .27^*^ | .36^*^ |
| ^§^ *Pearson correlation coefficients are given*.  *^*^p <.0001*  ^†^ *p<.05* | | | | | | | | | | |
