# Supplementary material for: Adolescents' wellbeing and functioning: relationships with parents' subjective general physical and mental health
Source: Health Qual Life Outcomes. 2009 Dec 15;7:100. doi: 10.1186/1477-7525-7-100 (PMC2804705; doi:10.1186/1477-7525-7-100)
Supplement: Additional file 2 — Multivariate analyses of the KIDSCREEN-52 questionnaire's ten dimensions [file 1477-7525-7-100-S2.DOC]

**Multivariate analyses of the KIDSCREEN-52 questionnaire’s ten dimensions**

| **Dimensions of Adolescents’ HRQoL: *β* (95% C.I.)** | | | | | | | | | | |
| --- | --- | --- | --- | --- | --- | --- | --- | --- | --- | --- |
| **Variables** | Physical Well-being  *(N=950)* | Psychological Well-being  *(N=959)* | Moods and Emotions  *(N=947)* | Self Perception  *(N=958)* | Autonomy  *(N=1 006)* | Parent Relations and Home Life  *(N=940)* | Social Support and Peers  *(N=1 152*) | School Environment  *(N=930)* | Social Acceptance and Bullying *(N=1 025)* | Financial Resources  *(N=903)* |
| PCS | NI | NI | NI | .32^†^  (.12, .51) | NI | NI | NI | NI | NI | NI |
| MCS | .26*  (.13, .39) | .28*  (.15, .40) | .20^†^  (.08, .31) | NI | NI | .20^†^  (.07, .32) | NI | .24^†^  (.12, .36) | NI | .36*  (.20, .52) |
| Age | -2.27*  (-2.93, -1.62) | -1.99*  (-2.64,-1.35) | -2.32*  (-2.92, -1.72) | -.74^†^  (-1.46, .-02) | -2.43*  (-3.18, -1.68) | -1.92*  (-2.58, -1.27) | NI | -2.76*  (-3.40, -2.12) | NI | NI |
| *Gender* |  |  |  |  |  |  |  |  |  |  |
| Female | Ref | Ref | Ref | Ref | Ref | Ref | Ref | NI | Ref | NI |
| Male | 8.17^*^  (5.93, 10.41) | 5.09^*^  (2.88, 7.31) | 7.01^*^  (4.95, 9.07) | 10.57^*^  (8.10, 13.04) | 7.00^*^  (4.40, 9.59) | 4.00^†^  (1.74, 6.26) | 2.93^†^  (.65, 5.21) |  | -1.97^†^  (-3.63, -.31) |  |
| *Family affluence scale* | | |  |  |  |  |  |  |  |  |
| Low | NI | NI | NI | NI | NI | NI | NI | NI | NI | Ref |
| Medium |  |  |  |  |  |  |  |  |  | 10.70^*^  (7.60, 13.79) |
| High |  |  |  |  |  |  |  |  |  | 16.93*  (12.88, 20.99) |
| *Results from CSHCN screener* | | |  |  |  |  |  |  |  |  |
| Negative | Ref | NI | NI | NI | NI | NI | NI | Ref | Ref | NI |
| Positive | -6.54^†^  (-12.71,-0.38) |  |  |  |  |  |  | -7.06^†^  (-12.98,-1.14) | -7.48^†^  (-12.02,-2.94) |  |
| OSLO social support | 2.49*  (1.89, 3.08) | 3.50*  (2.91, 4.08) | 3.59*  (3.05, 4.13) | 3.56*  (2.91, 4.21) | 2.98*  (2.29, 3.66) | 4.73*  (4.14, 5.33) | 4.78*  (4.18, 5.38) | 2.93*  (2.35, 3.51) | 2.23*  (1.79, 2.67) | 4.02*  (3.26, 4.78) |
| R^2^-adjusted | 0.19 | 0.21 | 0.26 | 0.18 | 0.12 | 0.27 | 0.18 | 0.19 | 0.10 | 0.22 |
| *NI indicates variables that are not included in the model for a specific outcome measure.*  *Ref indicates the reference category of non-continuous variables.*  ** p <.0001*  *^†^ p <.05* | | | | | | | | | | |
